# The Eclectic Practitioners, or the So-called Practical Men

**Published:** 1848-06

**Authors:** 


*The Eclectic Practitioners, or the so-called Practical Men.*—There are medical men in high positions, greatly occupied with numerous patients, who, from a want of study, of intelligence, or of time, from a natural indolence, or from being too old to master recent important improvements, affect a supreme disdain for every thing that concerns doctrine or generalization, either physiological or philosophical. They call themselves PRACTICAL men, and speak ironically of *theorists—men of science* or *of the closet*, such who labour most for the advancement of medical science, and whose knowledge crushes and confounds them. These so-called practical men are those who have no doctrine and no general principles, who gather together ready made formulæ and isolated cases, without any kind of scientific discernment. The only medicine they study is that contained in small books of prescriptions, published in 18mo. which they carry in their pocket, and know by heart. We have frequently had occasion to remark that a practical man, that is, a man who boasts of knowing nothing of scientific medicine, is a medical machine inferior intellectually to a master mason, a locksmith, or a cabinet-maker, for these have principles and a sort of doctrine which they apply in their business. They were appreciated in a like manner by a learned individual whose authority no one could doubt, and who said,—" The true eclectic works without conviction, without principle, without idea. He is continually enlarging his circle, in order to enclose within it facts of the most contradictory nature—they sacrifice in a sort to every god, and create a kind of scientific pantheism, not less fatal to true science than pantheism, properly so-called, is to true religion.—*Professor Cruveilhier's Address to the Anatomical Society.*

